# Aquatic Bird Bornavirus 1 in Wild Geese, Denmark

**DOI:** 10.3201/eid2112.150650

**Published:** 2015-12

**Authors:** Anders F. Thomsen, Jesper B. Nielsen, Charlotte K. Hjulsager, Mariann Chriél, Dale A. Smith, Mads F. Bertelsen

**Affiliations:** University of Copenhagen, Copenhagen (A.F. Thomsen, J.B. Nielsen);; Copenhagen Zoo, Copenhagen, Denmark (A.F. Thomsen, J.B. Nielsen, M.F. Bertelsen);; Technical University of Denmark, Copenhagen (C.K. Hjulsager, M. Chriél);; University of Guelph, Guelph, Ontario, Canada (D.A. Smith)

**Keywords:** Bornaviridae, geese, Denmark, Europe, zoonoses, viruses, bornaviruses, aquatic bird bornavirus 1, ABBV-1

## Abstract

To investigate aquatic bird bornavirus 1 in Europe, we examined 333 brains from hunter-killed geese in Denmark in 2014. Seven samples were positive by reverse transcription PCR and were 98.2%–99.8% identical; they were also 97.4%–98.1% identical to reference strains of aquatic bird bornavirus 1 from geese in North America.

Avian bornaviruses were first identified as the probable causative agent of proventricular dilatation disease in parrots in 2008 (*1*,*2*). Eight psittacine viruses and 5 passerine viruses have since been described (*3*,*4*). In 2009, aquatic bird bornavirus 1 was detected in free-ranging Canada geese (*Branta canadensis*) and trumpeter swans (*Cygnus buccinator*) in Ontario, Canada (*5*). Subsequently, this bornavirus has been detected across North America in at least 15 species of free-ranging wild birds (*6*,*7*). Initially designated as ABV-CG due to the high prevalence in Canada geese, the virus has been renamed aquatic bird bornavirus 1 (ABBV-1) in a reorganization of the taxonomy of the *Bornaviridae* (*4*). A second waterfowl-associated virus (ABBV-2) was isolated from ducks in North America in 2014 (*8*). Despite the fact that North American and European waterfowl are known to share breeding grounds in the Arctic, avian bornaviruses had not been detected in wild birds outside North America. The purpose of this study was to investigate the presence of aquatic bird bornavirus 1 in wild waterfowl in Denmark.

## The Study

By using real-time reverse transcription PCR (RT-PCR), we screened brain tissue from 333 hunter-killed geese from 9 locations in Denmark (Table; Technical Appendix Figure), collected during November and December 2014. Each screened sample consisted of pooled tissue from 5 individual birds, of the same species, collected at the same location. Primers and probe targeting the matrix (M) gene specific for ABBV-1 (*9*) were used. RNA was purified from 35 mg of pooled or individual brain samples with the QIAGEN RNeasy Mini Kit (QIAGEN, Copenhagen, Denmark), according to instructions from the supplier. Each PCR reaction contained 5 µL RNA; 1× RT-PCR buffer (AgPath-ID One-Step RT-PCR Kit; Life Technologies, Naerum, Denmark); 0.5 µmol/L of each primer, 0.25 µmol/L FAM-BHQ-1 labeled probe; and 1× RT-PCR enzyme mix in a total volume of 25 µL. The reactions were run on Rotor-Gene Q (QIAGEN) at 45°C, for 600 s, 95°C for 600 s, followed by 45 cycles of 94°C for 5 s and 60°C for 60 s. Data were analyzed with Rotor-Gene Q Series Software version 2.3.1 (QIAGEN). Parameters were adjusted as follows: dynamic tube, on; slope correct, on; ignore first cycle, 1; outlier removal, 10%; threshold fixed, 0.01. All other settings were default.

**Table:** Number and species of wild geese tested for ABBV-1 in 9 locations, Denmark, 2014*

Species	No. positive/no. tested (%)									
	Randbøl	Wadden Sea	Lolland	Skelby	Værnengene	Mandø	Møn	Skjern Enge	Western coast of Jutland	All locations
Greylag goose	0/8	1†/37 (2.7)	1‡/41 (2.4)	0/2	0/3	–	1§/10 (1)	1¶/28 (3.6)	1#/6 (16.7)	5/135 (3.7)
Pink-footed goose	–	–	–	0/1	0/8	–	–	1**/41 (2.4)	0/53	1/103 (1.0)
Barnacle goose	–	–	–	0/3	–	1††/47 (2.1)	–	–	–	1/50 (0.2)
Taiga bean goose	–	–	0/7	–	–	–	–	–	–	0/7
Tundra bean goose	–	–	0/7	0/1	–	–	–	–	–	0/8
White-fronted goose	–	–	0/6	0/1	–	–	–	0/1	0/8	0/16
Canada goose	–	–	–	–	–	–	0/13	–	–	0/13
Hybrid	–	–	–	–	–	–	0/1	–	–	0/1
All species	0/8	1/37 (2.7)	1/61 (1.6)	0/8	0/11	1/47 (2.1)	1/24 (4.1)	2/70 (2.9)	1/67 (1.5)	7/333 (2.1)

*ABBV-1, aquatic bird bornavirus 1. Dashes indicate none tested. †GenBank accession no. KR075030. ‡GenBank accession no. KR075031. §GenBank accession no. KR075033. ¶GenBank accession no. KR075035. #GenBank accession no. KR075036. **GenBank accession no. KR075034. †† GenBank accession no. KR075032.

Samples from birds in positive pools were purified and tested individually by quantitative -PCR and confirmed by endpoint conventional RT-PCR with a 2.200-bp amplicon covering the nucleocapsid (N), X protein, phosphoprotein (P), and partial M genes. Primers were previously published (*7*). Each reaction contained 1× buffer, 1.2 µmol/L of each primer, 0.4 µmol/L dNTP mix, 0.4 µmol/L enzyme mix (QIAGEN OneStep RT-PCR Kit; QIAGEN), and 5 µL purified RNA, in a total volume of 25 µL. Amplification was performed on a T3 PCR machine (Biometra, Fredensborg, Denmark) with cycling conditions 30 min at 50°C, 15 min at 95°C, and 120 s at 94°C, followed by 35 cycles of 30 s at 94°C, 30 s at 50°C, and 150 s at 68°C, and a final elongation at 68°C. Products were analyzed on 0.8% agarose E-Gels (Invitrogen, Naerum, Denmark) and verified by Sanger sequencing (LGC Genomics, GmbH, Berlin, Germany) with primers previously published (*1*) and the following primers: 5′-CAGCTCCAGTAAGGTGAGTTG-3′, 5′-CGCCGACTAGTGGACAGCCC-3′, and 5′-CTGCGGCATTCTACTGGAG-3′. Sequence data were edited with CLC Main Workbench 7.0 (CLC bio, QIAGEN, Aarhus, Denmark).

Seven ABBV-1–positive brain samples from individual birds were identified (Table). These samples were from 3 species of geese originating in 6 of 9 locations sampled. The N, X, P, and partial M fragment sequences were aligned, and a neighbor-joining tree with 1,000 bootstrap replicates was constructed with the CLC software and edited by using FigTree version 1.4.2 (http://tree.bio.ed.ac.uk/) (Figure). The European sequences from this study clustered together with North American ABBV-1, and a pairwise comparison in CLC software showed 98.2% to 99.8% identity among the 7 sequences and 97.4% to 98.1% identity to the reference strain of ABBV-1 (GenBank accession no. KF578398).

**Figure:**
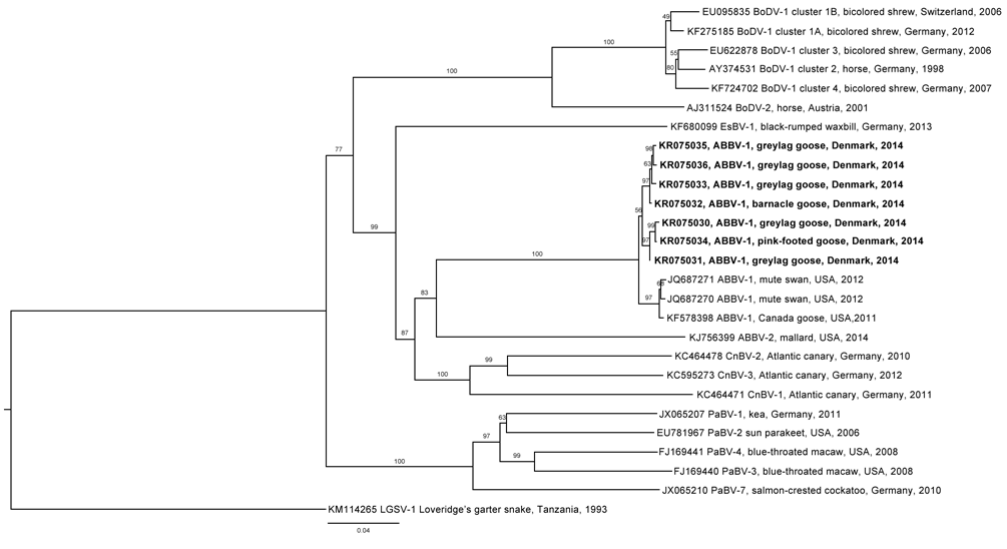
Phylogenetic tree comparing aquatic bird bornavirus 1 sequences obtained from waterfowl in Europe with selected bornavirus sequences from GenBank. Bold indicates viruses isolated in this study. Numbers along branches indicate bootstrap values. Scale bar indicates nucleotide substitutions per site.

The 2.1% (95% CI 0.6%–3.6%) prevalence of ABBV-1 in wild geese in Denmark is considerably lower than the prevalences reported in North America, which average 10%–30% but in some studies have exceeded 50% (*6*,*7*,*9*,*10*). In 1 study, the prevalence of ABBV-1 was higher in stable nonmigrating populations of Canada geese than in migratory birds, suggesting that prevalence may vary with population density and intensity or duration of use of geographic locations. In the investigation described here, we surveyed only a limited number of species previously identified as positive for ABBV-1 but identified 3 novel host species. Thus, the low prevalence found in geese in Denmark might be due to the sampling of a high proportion of transient migratory and apparently healthy birds as well as a possible variation in species susceptibility.

The finding of ABBV-1 in migratory waterfowl in Denmark suggests that the virus is widespread in waterfowl populations in Europe, but further investigation is needed to verify this claim. Pink-footed geese (*Anser brachyrhynchus*) and greylag geese (*A. anser*) migrate through the western part of Europe from Svalbard, Norway, to Spain. Barnacle geese (*A. leucopsis*) migrate from wintering grounds in the Wadden Sea area to northern Scandinavia and Russia. Because migrating waterfowl often gather in flocks of mixed species, transmission of pathogens between species is possible, and even likely, on the basis of our findings of nearly identical (99.7%) sequences in 1 pink-footed goose and in 1 greylag goose. The origin of ABBV-1 cannot be determined from this study, but the presence of highly homologous viruses in North America and Europe promotes speculation on possible transmission routes between these continents. Avian populations in Greenland could be the link between American and European flocks; the country is host to large breeding populations of geese that winter in both North America and in Europe.

The results here do not allow the clinical implications of ABBV-1 infections in waterfowl in Denmark to be determined, because none of the sampled geese were reported to be ill, and only goose heads were examined in the study. In North America, birds infected with ABBV-1 have exhibited nonsuppurative inflammation of the central, peripheral and autonomous nervous systems and associated neurologic and gastrointestinal clinical signs, including proventricular stasis.

## Conclusions

This study identifies ABBV-1 in wild geese in Europe; phylogenetic analyses demonstrated that the sequences from our investigation cluster with those from North America in the waterbird-1 cluster. The barnacle goose, greylag goose, and pink-footed goose were added to the list of waterfowl known to be hosts of ABBV-1. On the basis of the migration patterns of the affected species, we propose that the virus is distributed widely in Europe, but further investigation is needed to determine the validity of this hypothesis.

Technical Appendix The technical appendix consists of a figure showing the geographic origins of wild geese tested for aquatic bird bornavirus 1 in Denmark.

## References

[1] Kistler AL, Gancz A, Clubb S, Skewes-Cox P, Fischer K, Sorber K, Recovery of divergent avian bornaviruses from cases of proventricular dilatation disease: identification of a candidate etiologic agent. Virol J. 2008;5:88. 10.1186/1743-422X-5-88 18671869 PMC2546392

[2] Honkavuori KS, Shivaprasad HL, Williams BL, Quan P, Hornig M, Street C, Novel Borna virus in psittacine birds with proventricular dilatation disease. Emerg Infect Dis. 2008;14:1883–6. 10.3201/eid1412.080984 19046511 PMC2634650

[3] Philadelpho NA, Rubbenstroth D, Guimarães MB, Ferreira AJP. Survey of bornaviruses in pet psittacines in Brazil reveals a novel parrot bornavirus. Vet Microbiol. 2014;174:584–90. 10.1016/j.vetmic.2014.10.020 25465670

[4] Kuhn JH, Dürrwald R, Bào Y, Briese T, Carbone K, Clawson AN, Taxonomic reorganization of the family *Bornaviridae.* Arch Virol. 2015;160:621–32. 10.1007/s00705-014-2276-z 25449305 PMC4315759

[5] Delnatte P, Berkvens C, Kummrow M, Smith DA, Campbell D, Crawshaw G, New genotype of avian bornavirus in wild geese and trumpeter swans in Canada. Vet Rec. Vet Rec. 2011;169:108. 10.1136/vr.d4620 21784813

[6] Payne SL, Delnatte P, Guo J, Heatley JJ, Tizard I, Smith DA. Birds and bornaviruses. Anim Health Res Rev. 2012;13:145–56. 10.1017/S1466252312000205 23253163

[7] Guo JH, Covaleda L, Heatley JJ, Baroch JA, Tizard I, Payne SL. Widespread avian bornavirus infection in mute swans in the Northeast United States. Vet Med Res Rep. 2012;3:49–52. 10.2147/VMRR.S33353 PMC6065583 30155433

[8] Guo J, Shivaprasad H, Rech RR, Heatley JJ, Tizard IR, Payne SL. Characterization of a new genotype of avian bornavirus from wild ducks. Virol J. 2014;11:197. 10.1186/s12985-014-0197-9 25408146 PMC4239314

[9] Delnatte P, Ojkic D, DeLay J, Campbell D, Crawshaw G, Smith DA. Pathology and diagnosis of avian bornavirus infection in wild Canada geese (*Branta canadensis*), trumpeter swans (*Cygnus buccinator*) and mute swans (*Cygnus olor*) in Canada: a retrospective study. Avian Pathol. 2013;42:114–28. 10.1080/03079457.2013.769669 23581438

[10] Payne S, Covaleda L, Jianhua G, Swafford S, Baroch J, Ferro PJ, et al. Detection and characterization of a distinct bornavirus lineage from healthy Canada geese (*Branta canadensis*). J Virol. 2011;85:12053-6. 10.1128/JVI.05700-11 PMC3209299 21900161

