# Supplementary material for: Aquatic Bird Bornavirus 1 in Wild Geese, Denmark
Source: Emerg Infect Dis. 2015 Dec;21(12):2201–3. doi: 10.3201/eid2112.150650 (PMC4672415; doi:10.3201/eid2112.150650)
Supplement: Technical Appendix — The technical appendix consists of a figure showing the geographic origins of wild geese tested for aquatic bird bornavirus 1 in Denmark. [file 15-0650-Techapp-s1.pdf]

Article DOI: http://dx.doi.org/10.3201/eid2112.150650

Aquatic Bird Bornavirus 1 in Wild Geese, Denmark

Technical Appendix

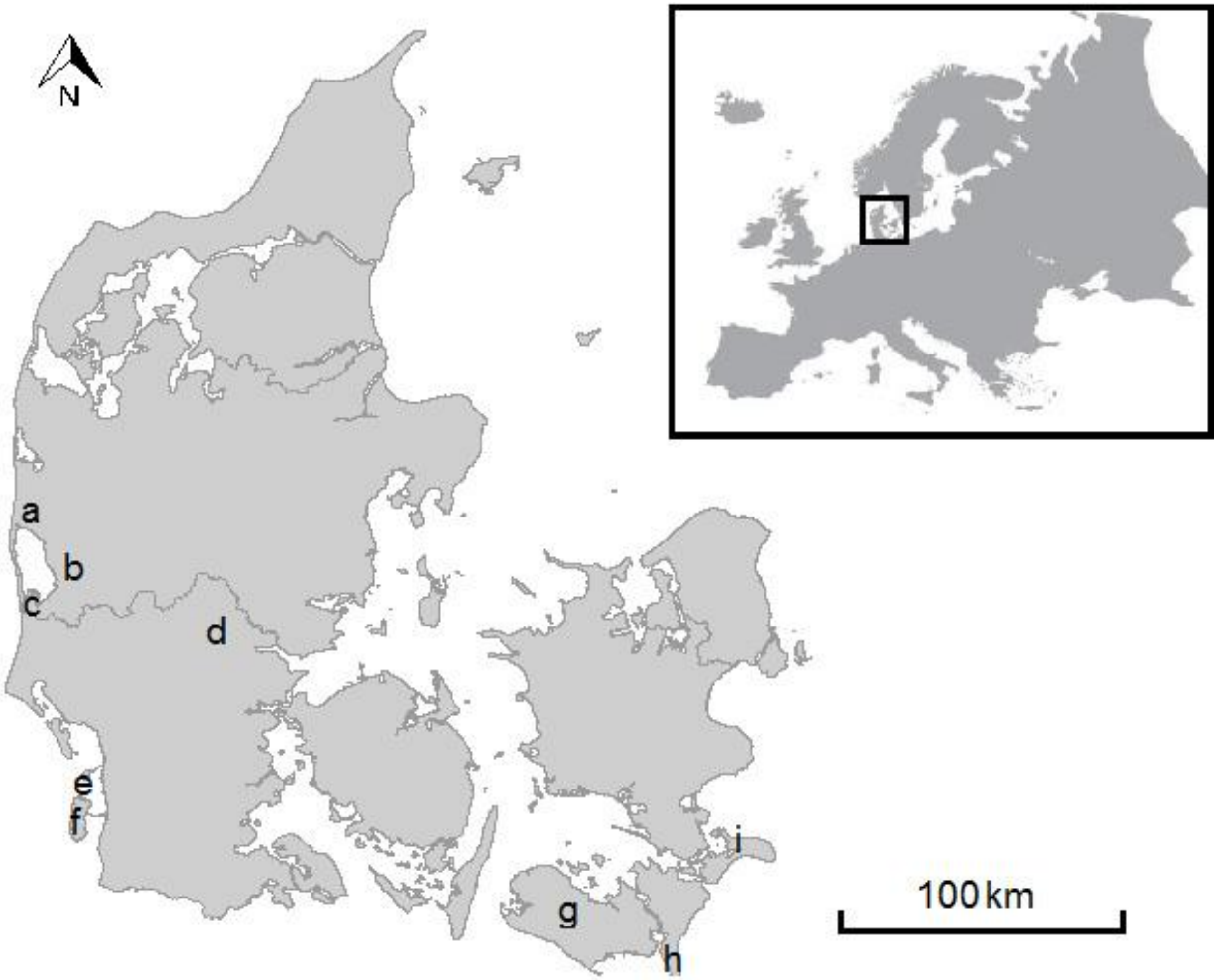

Technical Appendix Figure. Map of Denmark showing the geographic origins of wild geese tested for aquatic bird bornavirus 1 in 2014: a) west coast of Jutland; b) Skjern Enge; c) Værnengene; d) Randbøl; e) Mandø; f) Wadden Sea; g) Lolland; h) Skelby; j) Møn.
